# Detergent-Free Decellularization of Notochordal Cell-Derived Matrix Yields a Regenerative, Injectable, and Swellable Biomaterial

**DOI:** 10.1021/acsbiomaterials.2c00790

**Published:** 2022-08-09

**Authors:** Tara C. Schmitz, Marina van Doeselaar, Marianna A. Tryfonidou, Keita Ito

**Affiliations:** † Orthopaedic Biomechanics, Department of Biomedical Engineering, Eindhoven University of Technology, P.O. Box 513, Eindhoven 5600 MB, The Netherlands; ‡ Department of Clinical Sciences, Faculty of Veterinary Medicine, Utrecht University, Yalelaan 108, Utrecht 3584 CM, Netherlands

**Keywords:** decellularization, notochordal cell-derived matrix, intervertebral disc, nucleus pulposus, biomaterial, regeneration

## Abstract

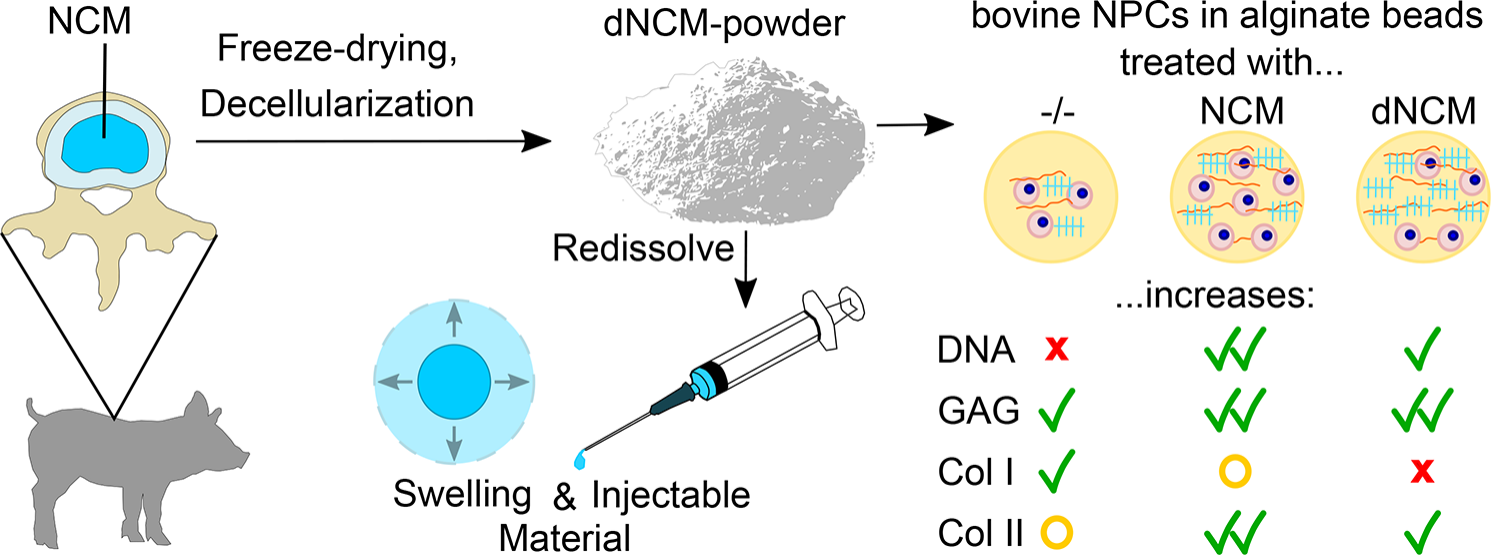

Porcine notochordal cell-derived matrix (NCM) has anti-inflammatory and regenerative effects on degenerated intervertebral discs. For its clinical use, safety must be assured. The porcine DNA is concerning because of (1) the transmission of endogenous retroviruses and (2) the inflammatory potential of cell-free DNA. Here, we present a simple, detergent-free protocol: tissue lyophilization lyses cells, and matrix integrity is preserved by limiting swelling during decellularization. DNA is digested quickly by a high nuclease concentration, followed by a short washout. Ninety-four percent of DNA was removed, and there was no loss of glycosaminoglycans or collagen. Forty-three percent of the total proteins remained in the decellularized NCM (dNCM). dNCM stimulated as much GAG production as NCM in nucleus pulposus cells but lost some anti-inflammatory effects. Reconstituted pulverized dNCM yielded a soft, shear-thinning biomaterial with a swelling ratio of 350% that also acted as an injectable cell carrier (cell viability >70%). dNCM can therefore be used as the basis for future biomaterials aimed at disc regeneration on a biological level and may restore joint mechanics by creating swelling pressure within the intervertebral disc.

## 1 Introduction

Intervertebral discs (IVDs) are an essential component of the functioning spine in vertebrates: they provide resistance to axial compression and allow for sufficient range of motion in 6 degrees-of-freedom. The nucleus pulposus (NP) comprises the center of the IVD. It is rich in glycosaminoglycans (GAGs) that create a swelling pressure by means of osmosis^1^ able to withstand the axial load within the spine.^2,3^ In the NP, nucleus pulposus cells (NPCs) are found in low numbers.^4,5^ Prior to adolescence, morphologically distinct vacuolated notochordal cells (NCs) can still be found in the human IVDs, which are thought to maintain the proliferative and maintenance capabilities of the NP.^6−9^ After childhood, these cells mostly disappear, and the inherent regenerative capacity of the IVD is greatly diminished.^10,11^ IVD degeneration slowly sets in, often culminating in lower back pain in adults many years later.^12^

NCs are thought to secrete a distinct set of >60 stimulating factors, leading to increased ECM production by NPCs,^7,8,13,14^ including connective tissue growth factor and transforming growth factor β.^15^ NC conditioned cell culture medium (NCCM) is able to inhibit senescence,^16^ as well as promote ECM production in NPCs.^7−9,17^ Porcine NCCM has been shown to outperform human NCCM,^9^ and porcine NC-derived matrix (NCM) thus presents an intriguing regenerative biomaterial for IVD degeneration therapy: it contains GAGs^18^ able to restore the swelling pressure within the IVD, as well as the growth factors excreted by the NCs able to stimulate biological regeneration.^8,19,20^

Extracellular DNA containing porcine endogenous retroviruses (PERVs) is problematic within the NCM.^21^ PERVs have been shown to infect human cells in vitro and thus pose a risk for patients.^22^ Furthermore, fragmented cell-free DNA may cause inflammation through several pathways^23,24^ and has been linked to chronic diseases like arthritis.^25^ Prior to utilization in a clinical setting, processing of the NCM to remove DNA is thus pertinent. Several decellularization protocols for porcine NP tissue have already been proposed, involving lengthy immersion into buffer with detergents.^26−28^ Detergents, however, have been shown to alter the composition and structure of decellularized tissues influencing the cell viability of infiltrating cells and are cytotoxic themselves.^29,30^ Crucially, detergents are known to deplete tissues of sulfated GAGs critical for swelling properties and potentially remove and/or denature bioactive proteins.^31−34^

Epitopes of Galα1-3-Galβ1-(3)4GlcNAc-R (α-Gal) are an important point in tissue transplantation, as they may be recognized by antibodies within the body, leading to graft rejection,^35^ but also remodeling.^36^ However, recent studies suggest a lack of α-Gal within the porcine NP.^37^ Thus, in this study, we chose to first focus on the removal of DNA to prevent transmission of endogenous viruses.

We therefore aimed to develop a simple detergent-free decellularization protocol for porcine NCM for the purpose of developing a bioactive functional biomaterial for cell delivery in IVD regeneration via injection. We examined the effect of decellularized NCM (dNCM) on bovine NPCs with respect to ECM production and anti-inflammatory properties and investigated dNCM’s biomaterial and cell-carrier properties.

## 2 Materials and Methods

If not otherwise stated, materials and chemicals were obtained from Sigma-Aldrich/Merck (Amsterdam, Netherlands).

### 2.1 Porcine NCM Isolation

Porcine spines (12 weeks old) were obtained from a local abattoir, according to local regulations. The IVDs were opened under aseptic conditions. Porcine NP tissue from IVDs of three spines was pooled into one batch, briefly mixed with a sterile weighing spoon, and then aliquoted into 1–2 g (wet weight) samples, yielding 100–200 mg of dry weight samples. Samples were frozen overnight at −80 °C before freezedrying in a lyophilizer (Labconco, Kansas City, US) for >72 h at ≤ −50 °C until completely dry (devitalized) to produce NCM and further decellularized to produce dNCM. A total of six batches were used in this study, half of each batch for NCM and the other half for dNCM, to obtain paired samples.

### 2.2 Decellularization of NCM to Obtain dNCM

Decellularization was performed under aseptic conditions. Lyophilized NCM samples were treated with 200 U/mL benzonase in 50 mM Tris-HCl buffer, pH 7.5, 2.5 mM MgCl_2_ at 0.01 mL buffer/mg dry weight tissue for 48 h at 37 °C on a roller at 2 rpm. The buffer volume was restricted to prevent GAG-mediated swelling and dissociation of the tissue during decellularization. Samples were then washed twice with 0.2 mL PBS/mg dry weight tissue for 30 min on a roller at 40 rpm. For easier buffer aspiration, samples were centrifuged at 1000 g for 5 min. As much PBS as possible was removed in between washes and prior to freezing and lyophilizing the samples for >72 h until completely dry. NCM and dNCM were pulverized using a mortar and pestle/microdismembrator (Sartorius, Goettingen, Germany), and then UV-sterilized in a Petri dish for 2 × 5 min, 1 × 10 min (stirring between) at 30 cm distance from a Philips TUVG30T8 UV lamp (Philips, Amsterdam, Netherlands).

### 2.3 Biochemical Content and Structural Changes of (d)NCM

Duplicate samples of NCM and dNCM were digested overnight at 60 °C using 140 mg/mL papain in 100 mM phosphate buffer, 5 mM l-cysteine, and 5 mM EDTA. The DNA concentration was determined using the Qubit DNA assay (Qubit dsDNA HS assay, Thermo-Fisher Scientific, Landsmeer, The Netherlands) following the manufacturer’s instructions. DNA fragment lengths were examined on a 1% agarose gel: 250 μL of digested sample was washed thrice with ultrapure water and then concentrated to 20 μL using ultracentrifugation filters with a 30 kDa molecular weight cutoff for gel electrophoresis^38^ (wash/concentration spins: 14 000 *g* for 10 min, recovery spin: 1000 *g* for 1 min). To visualize cell nuclei, we reconstituted NCM and dNCM powder to 10% w/v in PBS. Samples were then embedded into Tissue-Tek (Sakura, Finetek USA, Torrance, USA) on dry ice and thereafter stored at −20 °C. Sections were stained with 4′,6-diamidino-2-phenylindole (DAPI) (100 ng/mL in PBS) and imaged under an Axiovert 200 M microscope (Zeiss, Jena, Germany) (200 ms excitation time). GAG content was measured via the 1,9-dimethyl-methylene blue (DMMB) assay with shark chondroitin sulfate as reference standard.^39^ Hydroxyproline (HYP) content indicative of collagen was determined using the chloramine-T assay.^40^ The total protein content was measured on undigested, pulverized samples using the BCA assay (Thermo Fisher Scientific) and residual amount of benzonase was determined with a commercially available ELISA kit (Benzonase ELISA kit II). Samples were dissolved to 5 mg/mL in RIPA buffer with 1% cOmplete protease inhibitor (Roche, Mannheim, Germany) and incubated at 20 °C for 4 h, shaking at 300 rpm before centrifugation at 1000 *g* for 5 min. The supernatant was used for measurements of protein/benzonase content. All contents were normalized to tissue dry weight after decellularization. Structural changes throughout the decellularization process were visualized by alcian blue/haematoxylin staining of unprocessed NCM, dNCM after washing and 10% w/v reconstituted dNCM in PBS after second lyophilization and pulverization using bright-field microscopy (Axiovert Observer Z31, Zeiss, Jena, Germany).

### 2.4 Cytotoxicity of Benzonase

Neonatal human dermal fibroblasts (passage 6–8) (HDF106-05, ECACC, Salisbury, United Kingdom) were cultured in αMEM (Gibco, Landsmeer, The Netherlands) supplemented with 10% fetal bovine serum (FBS, Bovogen Biologicals, East Keilor, Australia), 1% penicillin/streptomycin (Gibco) and 1% l-glutamine (Gibco) at 37 °C with 21% O_2_ and 5% CO_2_. We used fibroblasts as a generic cell type, standing in for any potential cell type coming into contact with benzonase (NP cells, AF cells, transplanted cells, etc.). Cell viability in the presence of benzonase was tested using a 3-(4,5-dimethylthiazol-2-yl)-2,5-diphenyltetrazolium bromide (MTT, Molecular Probes, Landsmeer, The Netherlands) assay where cells were plated at 2.5 × 10^3^ cells/well in a 96-well plate. Benzonase concentrations were chosen to cover a wide range of concentrations, because the actual concentration of benzonase ending up in the IVD depends on the administered amount of dNCM as well as its eventual distribution volume. Cells were precultured for 48 h, before being incubated with various concentrations of benzonase for 48 h prior to MTT application (0.4 mg/mL in culture medium) for 75 min. Crystals were solubilized with 250 μL of DMSO/well for 30 min shaking at 300 rpm prior to absorbance measurements at 540 nm (690 nm reference) with a plate reader (Synergy HTX, BioTek, Winooski, United States). Samples were measured in triplicate and corrected for blank and background absorption. Untreated cells served as viable control; cells treated with 30% DMSO in culture medium instead of benzonase served as a nonviable control.

### 2.5 Bovine NP Cell Isolation and Alginate Bead Culture

Cell-free NCM when injected into the IVD has been shown to induce IVD regeneration by bioactively stimulating endogenous NPCs via soluble matrix-associated growth factors.^20,41^ Alginate beads are a common method to culture cartilaginous cells, like NPCs, in a 3D environment. We designed our experiment similarly to our previously published study for comparison:^42^ by adding pulverized dNCM into the medium, we allow its remaining soluble growth factors to diffuse into the alginate beads and investigate their effects onto encapsulated NPCs.

Bovine NP cells were harvested from mature bovine tails (24–36 months old) obtained from a local slaughterhouse according to local regulations. Discs were opened under aseptic conditions and the NP of each disc carefully removed. NP tissue was first digested in 0.1% Pronase (Roche) for 90 min at 37 °C, and then in 0.025% collagenase II (Worthington Biochemical Corporation, Lakewood, United States) for 16 h at 37 °C. Next, the cell suspension was filtered through a 70 μm pore-size cell strainer. For the alginate bead culture, cells were suspended in 1.2% alginate at 3 × 10^6^ cells/mL. The cell suspension was aspirated into a syringe through a blunt 18 gauge (G) needle and dropped through an 23 G needle into a 102 mM CaCl_2_-solution. Beads were then washed thrice with 0.9% NaCl-solution prior to culturing.

Alginate beads were cultured in low-glucose Dulbecco’s modified Eagle medium (DMEM) (Gibco) supplemented with 1% penicillin/streptomycin, 1% ITS-X (Gibco), 25 μg/mL ascorbic acid-2-phosphate, 40 μg/mL l-proline, and 1.25 mg/mL AlbuMAX (Roche). NCM or dNCM powder was added at 3 mg/mL to the culture medium. This concentration was chosen to mimic protein levels used in previous studies with NCM.^42^ Pro-inflammatory conditions were created by adding 5 ng/mL human IL-1β (Peprotech, Hamburg, Germany) during every media change throughout the entire culture duration as described before.^42^ Medium was changed 2–3 times per week. Beads were cultured for 28 days at 10% CO_2_ and 5% O_2_ at 37 °C. Medium was exchanged 2–3 times per week.

### 2.6 Bioactivity and Anti-inflammatory Properties of Decellularized NCM

ECM production by bovine NPCs within the alginate beads was assessed using the DNA, GAG, and HYP assays as mentioned before. Additionally, expression of key target genes (Table 1) relating to matrix anabolic/catabolic, and anti-inflammatory effects of NCM were monitored. Gene expression was normalized to HPRT using the 2^–ΔΔ*CT*^ method.^43^

**Table 1:** Key Genes Monitored for Change in Gene Expression in Presence/Absence of (d)NCM^a^

gene	accession number	primer pair sequences (5′ → 3′)	product size (bp)
HPRT	NM_001034035	FW: GAGGCATTGTGTCAGAGAGA	128
		RV: CTGTATTGAAAAGGAACTGTTGAC	
COL2A1	NM_001113224	FW: TGGCTGACCTGACCTGAC	187
		RV: GGGCGTTTGACTCACTCC	
COL1A2A	NM_174520	FW: TGAGAGAGGGGTTGTTGGAC	142
		RV: AGGTTCACCCTTCACACCTG	
ADAMTS-5	NM_001166515	FW: TCACTGCCTACTTAGCCCTGAA	125
		RV: GCTCCAACCGCTGTAGTTCAT	
MMP-13	NM_174389	FW: CTTGTTGCTGCCCATGAGTT	197
		RV: TTGTCTGGCGTTTTGGGATG	
ACAN	NM_173981	FW: CCAACGAAACCTATGACGTGTACT RV: GCACTCGTTGGCTGCCTC	107
IL-1β	NM_174093	FW: AGCATCCTTTCATTCATCTTTGAAG	88
		RV: GGGTGCGTCACACAGAAACTC	
IL-8	NM_173925.2	FW: TGCTTTTTTGTTTTCGGTTTTTG	71
		RV: AACAGGCACTCGGGAATCCT	
IL-6	NM_173923	FW: GGGCTCCCATGATTGTGGTA	69
		RV: GTGTGCCCAGTGGACAGGTT	
TNFα	NM_173966	FW: ACACCATGAGCACCAAAAGC	130
		RV: GCAACCAGGAGGAAGGAGAA	

a Annealing temperature of all primer pairs was 60 °C. HPRT: Hypoxanthine Phosphoribosyltransferase 1, COL2A1: collagen II alpha1 chain, COL1A2A: collagen I pro-alpha2 chain, IL-1*b*/6/8: interleukin-1*b*/6/8, TNFa: tumor necrosis factor α, ADAMTS-5: a disintegrin and metalloproteinase with thrombospondin motifs 5, MMP13: matrix metalloproteinase 13, FW: forward primer; RV: reverse primer; bp: base pairs.

### 2.7 Histology

Alginate beads were fixed for 1–2 h in 3.7% formalin with 102 mM CaCl_2_ and immersed in 30% sucrose for >4 h. Samples were then embedded as mentioned above. Sections were cut to 10 μm thickness with a cryotome (CM1950, Leica, Amsterdam, The Netherlands) and mounted onto Superfrost glass slides (Thermo Fisher Scientific). For details on detected structures and probes used, see Table 2.

**Table 2:** Probes Used for Immunohistochemistry

detection of	detecting molecule	supplier	concentration/dilution
nucleus	DAPI	Thermo Fisher Scientific	1 μg/mL
collagen I	rabbit anticollagen I	Abcam (ab34710)	1:200 (= 5 μg/mL) in 1% normal goat serum (Gibco)
	goat antirabbit Alexa-555	Molecular Probes (A21428)	1:200 (= 10 μg/mL) in PBS
collagen II	mouse IgG2a anticollagen II	Acris (AM00618PU-N)	1:200 (= 1 μg/mL) in 1% normal goat serum
	goat antimouse-IgG2a Alexa-555	Molecular Probes (A21137)	1:300 (= 6.67 μg/mL) in PBS

### 2.8 Swelling Capacity of dNCM

Ten percent w/v dNCM in 0.9% NaCl-solution was kept in media mimicking the healthy and degenerate discs (Table 3). The pH was adjusted to pH 7.1 and pH 6.8 for the healthy and degenerate conditions, respectively.

**Table 3:** Composition of Healthy and Degenerate Disc Environment-Mimicking Medium

component	healthy disc environment-mimicking medium	degenerate disc environment-mimicking medium	supplier
low glucose DMEM	4.99 g/500 mL	4.99 g/500 mL	Gibco
sodium bicarbonate	0.425 g/500 mL	0.213 g/500 mL	Sigma
*N*-methyl-glucamine HCl (NaCl homologue for adjusting medium osmolarity)	92.5 mmol/L (450 mOsm/kg)	47.5 mmol/L (350 mOsm/kg)	Sigma
penicillin/streptomycin	1% v/v	1% v/v	Gibco
l-ascorbic acid	25 μg/mL	25 μg/mL	Gibco
l-glutamine	1% v/v	1% v/v	Gibco
ITS-X	1% v/v	1% v/v	Gibco
l-proline	40 μg/mL	40 μg/mL	Sigma
albuMAX	1.25 mg/mL	1.25 mg/mL	Gibco

Gels were submerged in media and weighed at predetermined time points. Swelling was calculated relative to initial wet weight:
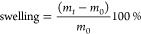
1with *m*_*t*_, wet weight at time point *t*, and *m*_0_, initial wet weight.

### 2.9 Rheological Properties of dNCM

The rheological properties of 10% dNCM in 0.9% NaCl-solution (*n* = 3) were measured in a parallel platen configuration (gap width 0.6 mm, diameter 25 mm), at 37 °C, using a rheometer (Ares 3000, TA Instruments, Asse, Belgium). Ten percent w/v dNCM was chosen to match NCM’s natural tissue composition, 0.9% NaCl was chosen to mimic clinical use. First a frequency sweep (0.1 rad/s to 100 rad/s, at 1% strain) was performed, followed by a strain sweep (0.1 to 100%, at 1 rad/s). The complex viscosity η* was obtained from the measured dynamic viscosities η′ and η′′ *via* the formula
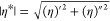
2

### 2.10 Injectability of dNCM

Injectability testing was conducted with human bone marrow-derived mesenchymal stromal cells (hBMSCs) (Lonza, Cohasset, United States).^44^ hBMSCs were cultured in high glucose DMEM (hgDMEM) supplemented with 10% FBS (Bovogen Biologicals), 1% penicillin/streptomycin, 1% nonessential amino acids, and 1 ng/mL basic fibroblast growth factor (bFGF, Peprotech) and passage 6 was used for the experiment. Reconstituted dNCM in 0.9% NaCl solution was combined with hBMSCs to yield final concentrations of 10% dNCM with 1, 5, and 10 million cells/mL. Samples were aspirated through an 18G blunt needle into a sterile syringe and ejected through a 27G needle into a dialysis membrane tube (15 kDa MWCO, Carl Roth). Samples were incubated for 24 h in hgDMEM (Gibco) with 10% FBS (Gibco), 1% penicillin/streptomycin, and 8.2% 20 kDa PEG to prevent swelling as described previously.^45^ A LIVE/DEAD staining was performed with calcein-AM/propidium iodide (1 μg/mL/10 μg/mL, respectively) (Invitrogen) for 1 h in serum-free hgDMEM. Samples were removed from the dialysis bags and transferred to a six-well plate and then covered with a coverslip. Pictures were acquired on a Apotome microscope (Zeiss). Cell viability was quantified using *ImageJ*. Samples were prepared in triplicate, with at least two fields examined per sample.

### 2.11 Statistics

Statistics were performed in *R* (v3.6.3). A Shapiro–Wilkes test was used to test for normality of distribution, and a Levene’s test for homogeneity of variances. A *t*-test was used to determine statistical differences in DNA content between NCM and dNCM. A Wilcoxon two-sample paired test was performed for differences in GAG, HYP, and protein content between NCM and dNCM. Differences in gene expression were analyzed with a Kruskal–Wallis test followed by a Dunn’s post hoc test. Alginate bead biochemical composition was investigated with a one-way ANOVA test and Tukey posthoc testing for normally distributed data, or a Kruskal–Wallis test followed by a Dunn’s post hoc test for non-normally distributed data. Viability after injection for different cell concentrations was analyzed with a one-way ANOVA test and Tukey posthoc testing. Differences in dNCM swelling dependent on media were investigated by a *t*-test. A cutoff of *p* < 0.05 was used to determine statistical significance.

## 3 Results

**Figure 1:**
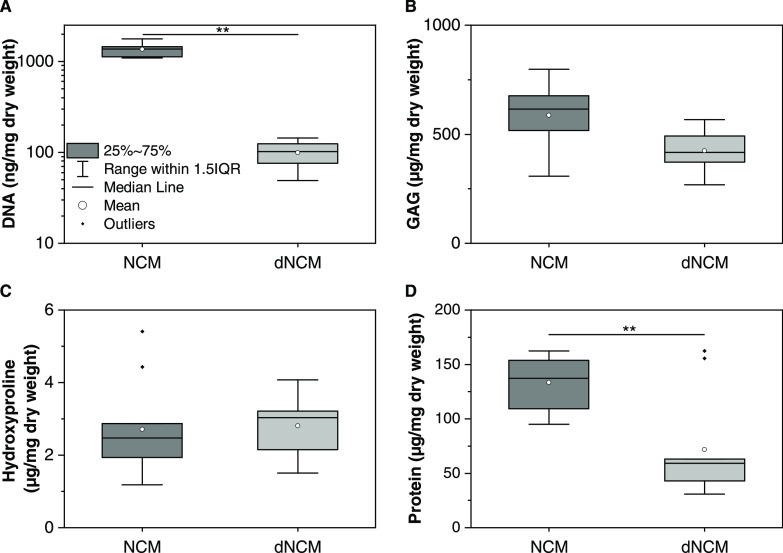
Tissue composition pre- and postdecellularization. GAG = glycosaminoglycans, NCM = notochordal cell-derived matrix, dNCM = decellularized notochordal cell-derived matrix. Six batches of three spines each were used; half of each batch was decellularized to obtain paired samples. Two samples from each batch were taken for analysis. Horizontal line indicates median, whiskers indicate 95% confidence intervals. *n* = 6, ** = *p* < 0.01. GAG, glycosaminoglycans; NCM, notochordal cell-derived matrix; dNCM, decellularized notochordal cell-derived matrix.

**Figure 2:**
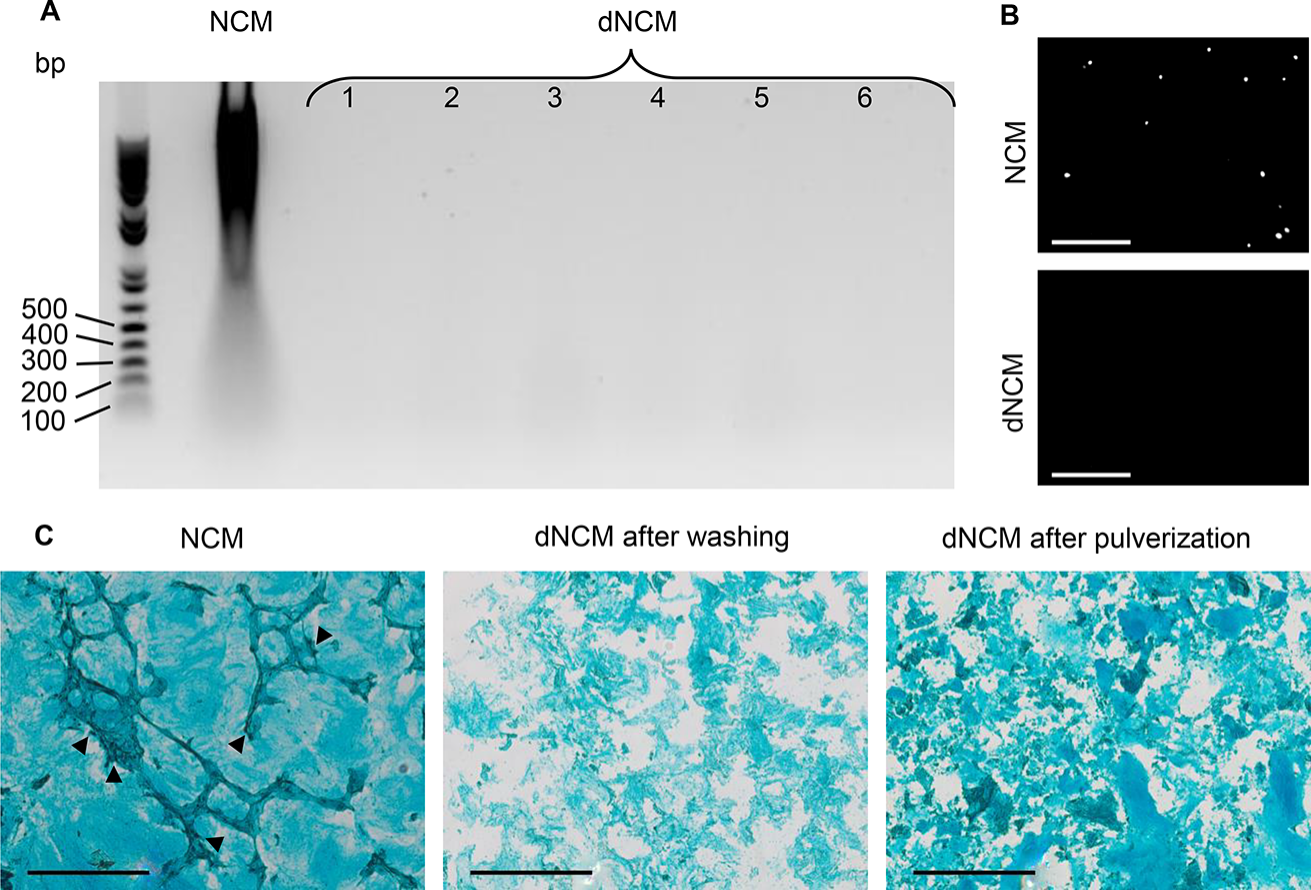
(A) DNA fragments in nondecellularized and decellularized notochordal cell-derived matrix (NCM and dNCM, respectively). Samples 1–6 exhibited a slight smear of DNA fragments <400 base pairs (bp) long. (B) DAPI staining of porcine nucleus pulposus and reconstituted dNCM. Scale bar: 100 μm, excitation time: 200 ms. (C) Alcian blue/haematoxylin staining of various stages during the decellularization process demonstrating removal of cell nuclei (arrow heads), and later loss of matrix and tissue structure after washing and pulverization of dNCM, respectively. Scale bar: 200 μm.

**Figure 3:**
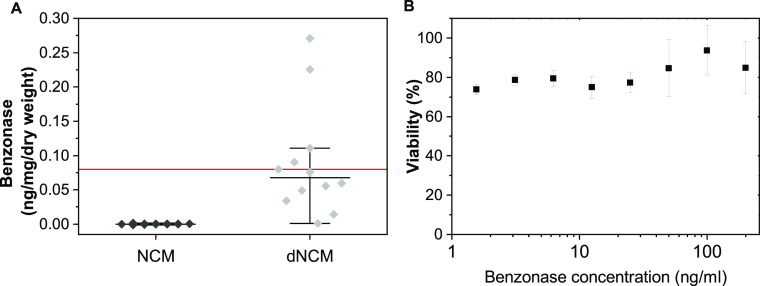
(A) Benzonase content in decellularized notochordal cell-derived matrix (dNCM) was <0.3 ng/mg dry weight and mostly below the detection limit (red line) of the ELISA kit used. Horizontal line indicates median, whiskers indicate 95% confidence intervals. *n* = 6, ** *p* < 0.01. (B) MTT assay results indicated cell viability is reduced in the presence of benzonase relative to untreated control, but does not scale with benzonase concentration. *n* = 3, average ± standard deviation plotted.

After decellularization, we found a reduction in DNA content of 93.9 ± 3.1% to ≈85 ng/mg tissue (Figure 1). DNA fragments were <400 bp in size (Figure 2A) and could not be detected microscopically after staining with DAPI, in contrast to nondecellularized NCM (Figure 2B). During the decellularization procedure, the initially cohesive tissue structure of NCM is fragmented (Figure 2C). Crucially, no statistically significant loss in GAG and HYP content was seen; median GAG content reduced from 615 μg/mg (interquartile range (IQR): 540–660 μg/mg) to 416 μg/mg (IQR: 384–468 μg/mg) (median 68% GAGs remain), whereas median HYP content slightly increased from 2.5 μg/mg (IQR: 2.0–2.8 μg/mg) to 3 μg/mg (IQR: 0.8–3.2 μg/mg). Median total protein content reduced from 137 μg/mg (IQR: 111–152 μg/mg) to 59 μg/mg (IQR: 45–63 μg/mg), i.e., 43% of protein content remained within the dNCM (Figure 1). Despite the high benzonase concentration used, most benzonase was removed from dNCM (Figure 3A). No loss in cell viability of fibroblasts was observed in the presence of benzonase concentrations 600× higher than that measured per milligram of dNCM (Figure 3B).

**Figure 4:**
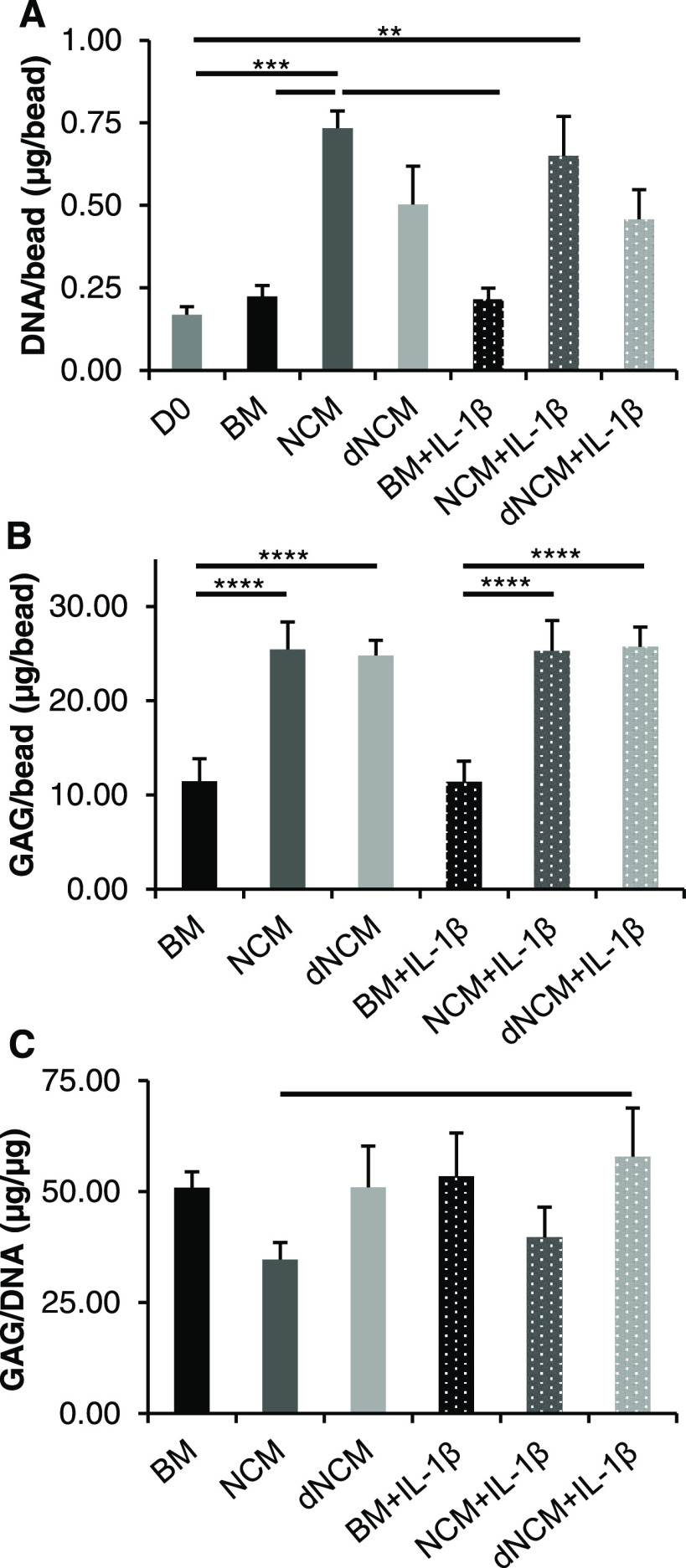
DNA and GAG content per alginate bead with encapsulated bovine NPCs. The effects of added NCM, dNCM (both supplemented at 3 mg dry weight/mL), and pro-inflammatory stimulus IL-1β (5 ng/mL) onto DNA and GAG levels were investigated. (A) Significant increases in DNA content were found for cells treated with NCM, but not dNCM after 28 days compared to day 0. (B) However, sGAG production by cells was equally increased in both treatment groups compared to base medium group after 28 days. (C) Relative increase in sGAG/DNA amounts highlights the promotion of proliferation over sGAG production in NCM-treated cells. No effect of IL-1β could be observed here. All *n* = 5, average ± standard deviation, — = *p* < 0.05, ** = *p* < 0.01, *** = *p* < 0.001, **** = *p* < 0.0001. BM, base medium; NCM, notochordal cell-derived matrix; dNCM, decellularized NCM; NPC, nucleus pulposus cell; GAG, glycosaminoglycan.

**Figure 5:**
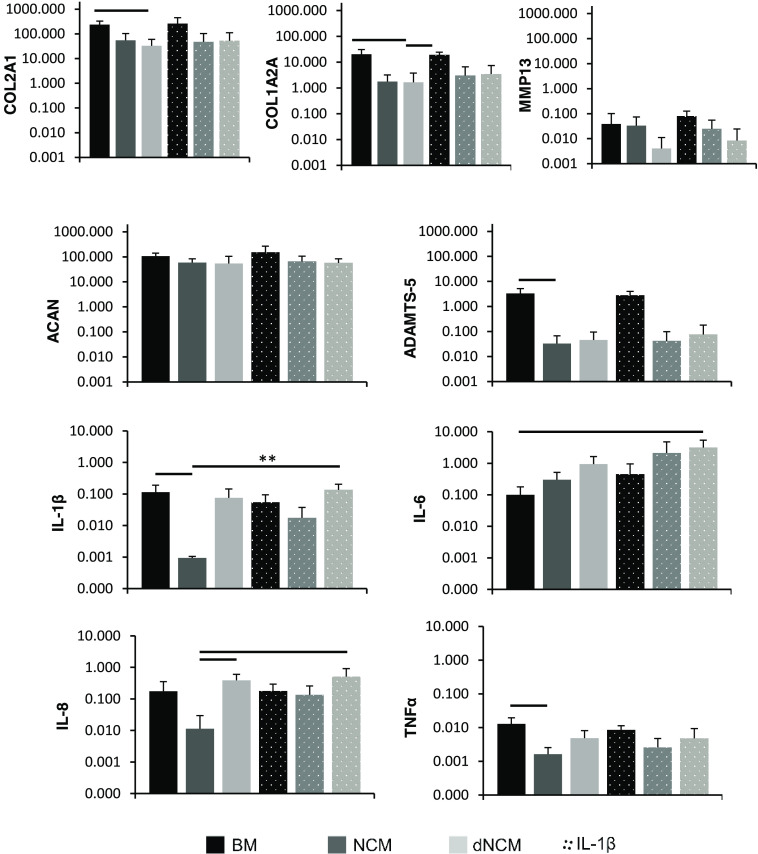
Gene expression of bNPCs in response to NCM/dNCM treatment after 28 days. Significant reduction of collagen I (COL1A2A) and collagen II (COL2A1) gene expression in cells treated with dNCM after 28 days was observed. Aggrecanase (ADAMTS-5) and collagenase (MMP13) gene expression tended to be lower in NCM- and dNCM-treated groups, whereas aggrecan (ACAN) gene expression remained unaffected. Significant reduction in IL-1β, IL-8, and TNFα expression was observed in the NCM-treated group after 4 weeks, but not in dNCM-treated groups. IL-6 tended to increase in the presence of NCM and dNCM, as well as IL-1β. *n* = 5, average ± standard deviation plotted. — = *p* < 0.05, ** = *p* < 0.01. NPC, nucleus pulposus cell; NCM, notochordal cell matrix; dNCM, decellularized NCM; BM, base medium.

The effect of NCM and dNCM was studied in conditions mimicking the normal and degenerate disc environment by stimulating bovine NP cells with IL-1β. In terms of the bioactivity of dNCM, we observed an overall anabolic response in response to NCM and dNCM with regard to protein content (Figure 4) and gene expression (Figure 5) in the 28-day culture with bovine NPCs encapsulated in alginate beads.

**Figure 6:**
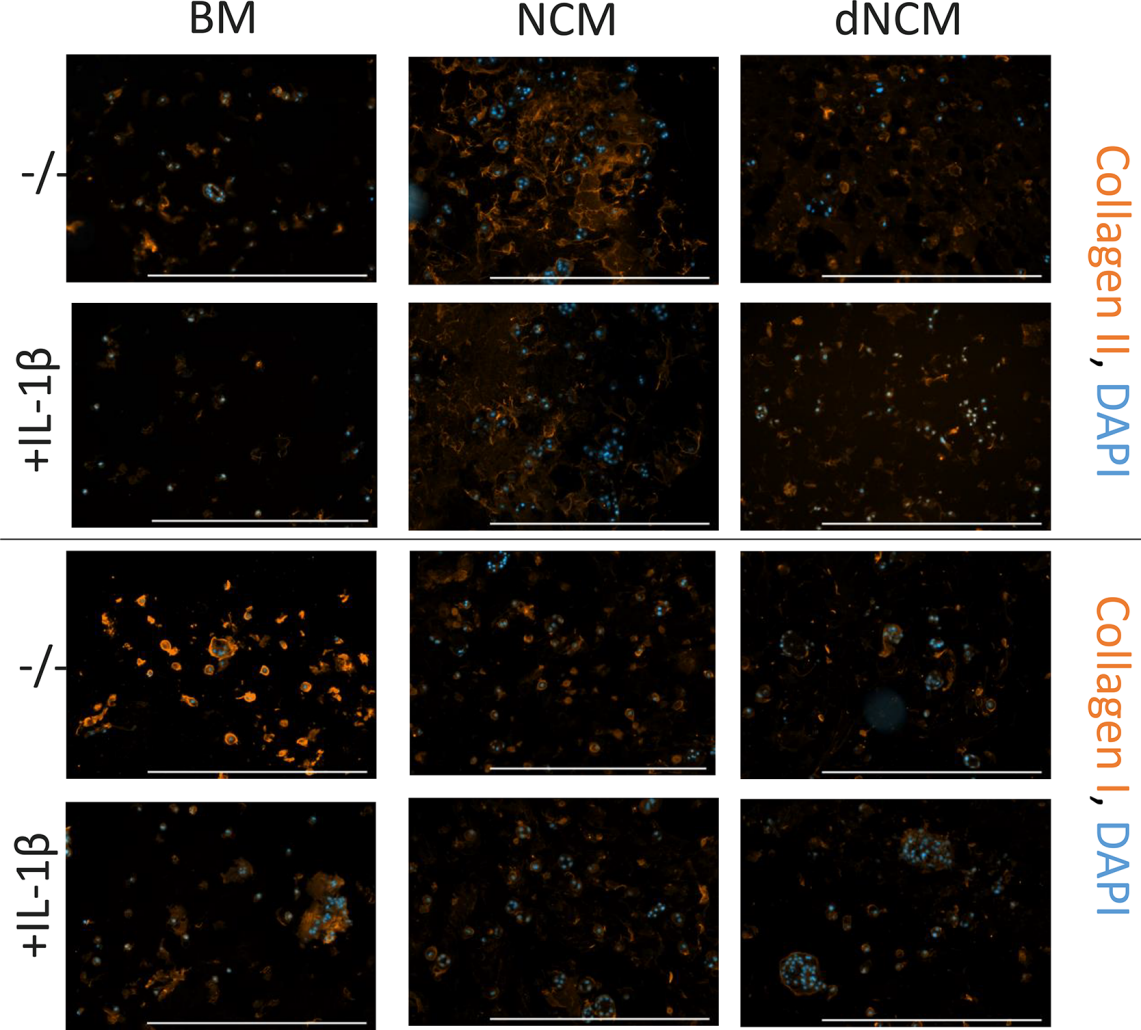
Bovine NPCs deposited different collagen types under different conditions. Collagen II deposition was enhanced in the presence of NCM, but not dNCM compared to untreated groups (top). Collagen I production was highest in untreated groups (bottom). IL-1β decreased collagen production across all groups. Scale bar: 500 μm. One second excitation time for collagen detection. BM, base medium; NCM, notochordal cell-derived matrix; dNCM, decellularized NCM.

No significant increase in DNA/bead in nontreated cells was seen over 28 days. NCM-treatment significantly increased DNA content/alginate bead with circa three times the amount of DNA/bead of nontreated cells and twice of dNCM-treated bovine NP cells. dNCM stimulated the same amount of sGAG production as NCM, both groups being significantly higher than nontreated cells. The GAG/DNA ratio tends to be higher for the BM and dNCM groups than the NCM group (Figure 4). At the gene expression level, collagen I and II expression were significantly reduced in dNCM-treated cells after 28 days compared to cells in base medium. On the protein level, however, dNCM-treated cells exhibited less collagen II deposition than NCM-treated groups, but still more than the base medium group (Figure 6). Collagen I was mostly present in the base medium group and was less visible in NCM- and dNCM-treated groups. Furthermore, aggrecan gene expression was not affected across all groups, whereas dNCM- and NCM-treated groups tended toward lowered expression of catabolic genes MMP13 and ADAMTS-5.

IL-1β treatment was provided to study the effect of dNCM under pro-inflammatory conditions. IL-1β did not affect DNA content nor GAG production (Figure 4) but decreased collagen type I and type II immunostaining intensity across groups (Figure 6), indicative of decreased deposition. In terms of NCM-dependent modulation of the inflammatory response, no significant differences in gene expression were found between nonstimulated cells and cells stimulated with IL-1β. NCM-treated groups exhibited significantly lower levels of IL-8 expression at day 28 compared to dNCM-treated cells (Figure 5). NCM significantly reduced the gene expression of IL-1β and TNFα compared to untreated groups. Stimulation with IL-1β in the medium abrogated any differences in interleukin expression between groups. Furthermore, a significant inflammatory effect of dNCM was seen only in combination with IL-1β-stimulation for IL-6 gene expression after 28 days.

**Figure 7:**
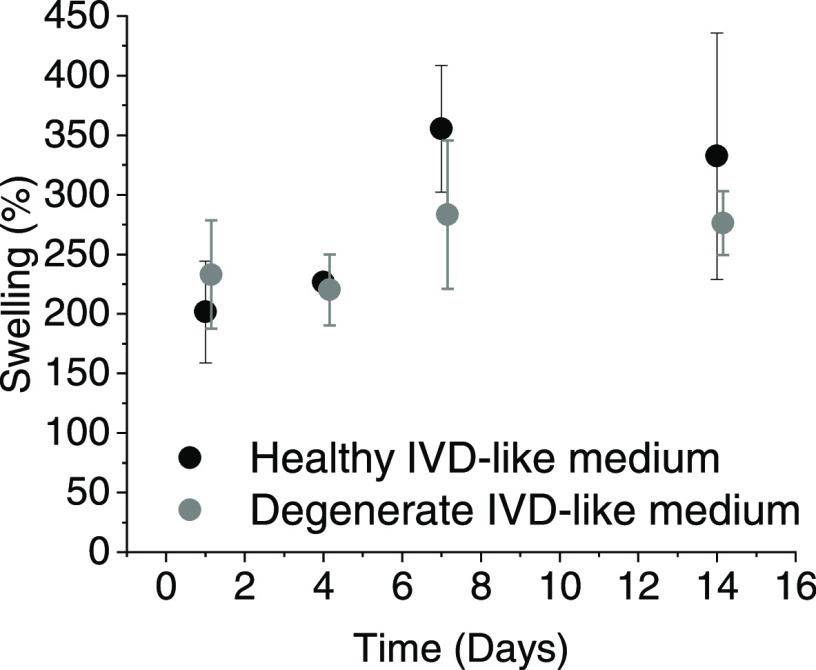
Hydration of 10% dNCM in 0.9% NaCl over time in media mimicking (non-)degenerative conditions found in the IVD (see Table 3 for media composition). *n* = 3, average ± standard deviation plotted.

Reconstituted dNCM swelled up to 300% relative to its starting weight (Figure 7) in free swelling conditions. Swelling capacity was unaffected by media mimicking the healthy and degenerate IVD environment.

**Figure 8:**
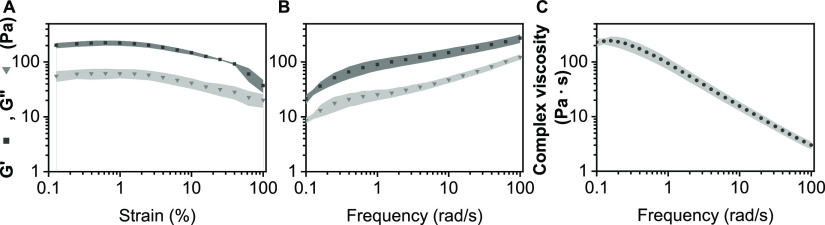
Behavior of 10% dNCM in saline solution under increasing (A) shear frequencies and (B) amplitudes followed that of a polymeric solution. (C) Viscosity decreased with increasing shear, indicating a shear-thinning solution. All *n* = 3, average ± standard deviation plotted.

**Figure 9:**
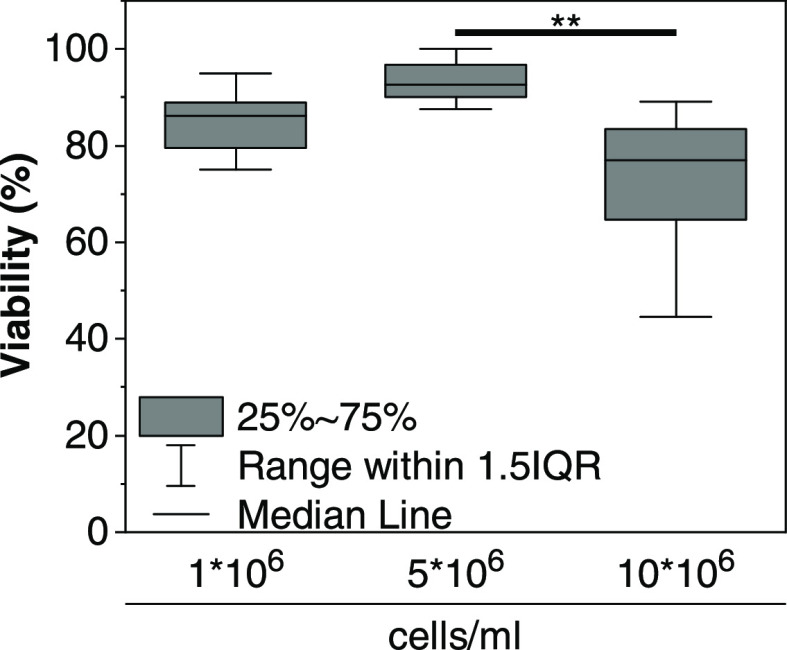
hBMSC viability in 10% dNCM 24 h postextrusion through a 27 G needle. *n* = 3, ** = *p* < 0.01.

We further found that the 10% w/v dNCM suspension had a low storage modulus (≈100–200 Pa) and decreasing viscosity with increasing shear (Figure 8). Injection of MSCs within dNCM through a clinically relevant 27-gauge needle did not adversely affect cell viability, with >77% median viability observed when injecting 10 × 10^6^ cells/mL, and >80% for lower cell concentrations (Figure 9).

## 4 Discussion

In this study, we aimed to create an effective detergent-free decellularization protocol for the notochordal-cell-rich NP maintaining its bioactive effects. In contrast to previous protocols, we did not aim to preserve the tissue structure and create a scaffold for tissue engineering purposes, but aimed to create a bioactive NCM-derived injectable material that preserves GAGs and proteins and may act as a cell vehicle for further disc regeneration approaches. Previously published protocols for decellularization of the porcine NP rely on immersion and perfusion of the isolated tissue in detergent-containing buffers to lyse cells. In doing so, however, the tissue may lose its integrity^26^ and some components may be lost over time, the amount lost depending on the specific protocol used.

As an alternative cell-lysis method, we lyophilized the tissue. To preserve as much tissue as possible, we prevented uncontrolled swelling of the tissue in buffer limiting tissue dissociation. To this end, we restricted the buffer volume to 0.01 mL/mg of tissue dry weight, effectively creating an incompletely swollen gel during the decellularization process. Similar to a recent previously published protocol,^28,37^ with our protocol, overall GAG and collagen content were not of statistically significant difference with decellularization despite differences in median values. A high GAG/collagen ratio of ≈27 ± 5 is characteristic for NCM^9^ and was only slightly reduced in dNCM to ≈21.3. At the same time, DNA content was reduced by 94%, to ≈85 ng/mg of dry weight.

In terms of residual DNA content after decellularization, amounts <50 ng of DNA/mg of tissue dry weight with <400 bp in length and lack of visibility in H&E or DAPI stains has been proposed as a positive outcome control measure.^46−48^ The measured total DNA content in the dNCM is higher at 85.3 ng/mg dry weight but is undetectable using DAPI and becomes detectable only after concentration on an agarose gel. Extracellular DNA has been implied in the severity of inflammation,^23−25^ but the amounts found in healthy individuals’ plasma vary greatly depending on the quantification method and lie either below or above the published limit for successful decellularization.^49^ As such, this proposed limit may not reflect a universally applicable goal,^38^ especially considering the immunoprotected state of the NP within the avascular IVD.^50^ Compared to the previously published protocols, we achieved a similar or greater reduction in DNA content when accounting for different normalization methods used (wet/dry weight). Considering the total genome length of PERVs at 9 kbp^51^ and individual gene length at >600 bp,^52^ the probability of infection by using decellularized NCM containing DNA fragments of <400 bp in length is minimal. The previously published studies^26−28^ did not concentrate samples to ensure visualization or indicate fragment sizes found or they investigated only the presence of larger fragment sizes, hindering comparisons in this aspect. Additionally, we measured the remaining trace amounts of nuclease in the decellularized tissue and evaluated its cytocompatibility. Less than 1% of initial benzonase input was detected in all samples after decellularization and was consistent, and 600× higher benzonase concentrations did not affect cell viability. The delivered amount to the disc depends on the formulation and volume of dNCM injected into the disc. Even levels 6× those found in 100 mg/mL dNCM (=10% w/v, mimicking water content in NCM) were tolerated. The effect of benzonase on the overall application of dNCM in potential clinical settings is anticipated to be minimal.

The overall loss of NCM’s tissue structure observed after washing reflects the loss in GAGs and increase in the porosity of the tissue. Once pulverized and condensed, GAG loss is somewhat compensated. Although tissue structure disruption is usually avoided for decellularization, in our application, we are more interested in injectability and maintaining the soluble bioactive factors of dNCM more so than its structure.

None of the previous studies decellularizing porcine NP examined total protein content or composition after decellularization, but still report enhanced matrix production by either NP cells^27^ or human adipose-derived stem cells.^53^ Collagen and GAGs are known to influence cell adhesion and behavior,^54,55^ but additionally, notochordal-cell-rich tissues like canine or porcine NCM exhibit many bioactive factors.^15,17^ These factors counteract NP degeneration, potentially by stimulating TGF-β-related pathways and preventing ECM degradation.^15^ According to our results, the total protein content does not reflect the combined proliferative and anabolic response of NP cells to dNCM treatment. With the protocol presented in this study, ≈43.2% of proteins remain in the dNCM, stimulating 71% as much DNA production, but comparable GAG production as NCM. Collagen deposition is affected differently between NCM- and dNCM-treated NPCs as well.

dNCM may therefore have an altered set of functional proteins responsible for lower proliferative stimulation compared to NCM. NCM is characterized by soluble and matrix-associated pelletable components.^17^ In our decellularization protocol, the matrix composition is kept intact while cells are destroyed via lyophilization. Larger molecules such as proteoglycans, GAGs, and collagen cannot easily diffuse out of the tissue during our protocol’s short washing step. However, NCM’s unbound smaller solutes would be free to diffuse out during enzyme incubation and the washout procedure. Proteins may be differentially washed out because of their physicochemical characteristics like size, hydrodynamic radius, charge, and protein–protein interactions,^56−58^ whereas susceptibility to UV degradation stems from their amino acid composition.^59,60^ Future proteomic studies may identify compositional changes between NCM and dNCM.

Compared to NCM, dNCM also may not possess an anti-inflammatory effect anymore. As previously published,^42^ NCM tended to lower IL-1β and TNFα gene expression in stimulated bovine NP cells (Figure 5) and in vivo in degenerate IVDs of experimental dogs.^20^ Cells treated with dNCM tend to express inflammatory markers on par with nontreated cells, except for a significant increase in IL-6 expression, a trend also seen in NCM-treated cells. Overall, the observed effect of IL-1β is small on gene and protein levels and point to a batch of bovine NPCs less responsive to the applied pro-inflammatory stimulus compared to our previous studies.^42^ A combination of pro-inflammatory mediators, e.g., TNF-α, could have been used to synergize with IL-1β and elicit a stronger and more robust cell responses.^61^ Importantly, although it does not shield NPCs from inflammatory stimuli, dNCM also does not cause additional harm to them. The overall stimulation of proliferation, collagen deposition, and GAG production renders dNCM a useful therapeutic agent for IVD regeneration. On the matrix level, GAG deposition by NPCs outweighs collagen II deposition in their importance to NP swelling pressure and restoration of biomechanics.^62^

We aimed to use the decellularized NCM as an injectable vehicle for IVD degeneration treatment. Although pepsin digestion and collagen refibrillation is commonly used to produce gels from decellularized matrices,^63^ translation to in-patient use for IVD degeneration treatment may be hindered by the reversible inactivation of the pepsin, which regains its activity in acidic environments,^64^ such as the degenerated IVD. Pepsin may be irreversibly inactivated at pH ≥8;^65^ however, this will affect not only pepsin but the decellularized ECM proteins and potential encapsulated cells as well. From our understanding, the increase to pH 8 did not occur when ECM-derived gels were prepared *via* pepsin digestion. Additionally, pepsin is a nonspecific protease^66^ and thus potentially reduces the remaining bioactivity of dNCM by digestion of the matrix-associated growth factors. Alternatively, reconstitution of dNCM to 10% w/v results in a suspension that could be described as a viscoelastic liquid.^67 ,68^ With a stiffness of ≈100–200 Pa, reconstituted dNCM falls within the stiffness range of decellularized ECM hydrogels obtained by collagen refibrillation.^63^ As such, this material does not exhibit meaningful load-bearing properties for use in the IVD.

Swelling is a central feature of the healthy NP biomechanics,^69^ and therefore NP biomaterials that aim to restore healthy motion segment mechanics in the spine.^70^ We tested the swelling capacity of dNCM by reconstituting it to 10% w/v in 0.9% NaCl in media mimicking the healthy and degenerate IVD environment. dNCM’s hydration capacity stems from the GAGs within^2^ and could contribute to reestablishing a swelling pressure within the disc. The predominant swelling mechanism for dNCM may stem from interaction of GAGs with water directly rather than the dissolved ions, as the swelling degree is not influenced by osmolarity of the medium (Figure 7).

Importantly, reconstituted dNCM exhibits a shear-thinning behavior (Figure 8), making it suitable for injection.^71^ Cell implantation for DDD treatment and IVD regeneration has been widely investigated and discussed.^72,73^ Mesenchymal stem cells (MSCs) have been previously tested for their therapeutic potential in IVD regeneration.^72^ We therefore investigated the potential of dNCM as a vehicle for cell transplantation by injection and found median viabilities >77% for administration of up to 10 × 10^6^ cells/mL, i.e., no drop in cell viability according to FDA standards.^74^ Higher cell concentrations experience higher shear stress during injection^75,76^ and competition for nutrients postinjection within the IVD.^72^ Lower cell concentrations are thus more favorable for clinical application but require highly potent cells for adequate novel matrix deposition and/or stimulation of resident NPCs.

While being a step closer to clinical translation, dNCM still has its limitations: IL-6 has been reported to induce IVD degeneration through YAP/β-catenin signaling.^77^ Previous studies already indicated an overall beneficial effect of NCM *in vivo*,^20^ despite the increase in gene expression of IL-6 observed in vitro in this and the previous study.^42^ Production of IL-6 on protein level in the presence of dNCM and its potential effect onto NPCs should therefore be investigated. For later clinical application, quantification of α-Gal epitopes within the dNCM is necessary because of its role in tissue remodeling and graft rejection.^78,79^ Adaptation of an ELISA for α-Gal-quantification^80^ remained unsuccessful on NCM tissue (data not shown). With the current protocol, α-Gal digestion can be achieved by the addition of α-galactosidase concurrently or sequential to tissue incubation with benzonase to yield an immunogenically better compatible material for use in human patients. We are currently exploring alternatives for detecting these glycan residues pre-/post-decellularization.

## 5 Conclusion

In this study, we developed a short, detergent-free, and easy protocol to decellularize porcine NCM. The anabolic stimulatory effect onto NPCs was kept after processing, and material properties show that reconstituted dNCM is a suitable vehicle for cell delivery into the disc, that may restore swelling pressure within the disc. dNCM can be further combined with load-bearing biomaterials for mechanical and biological restoration of the IVD. dNCM therefore holds great potential as a biomaterial for future IVD regeneration.

## References

[ref1] Urban J. P. G.; Maroudas A. Swelling of the Intervertebral Disc in Vitro. Connect. Tissue Res. 1981, 9 (1), 1–10. 10.3109/03008208109160234. 6456121

[ref2] Adams M. A.; Dolan P.; McNally D. S. The Internal Mechanical Functioning of Intervertebral Discs and Articular Cartilage, and Its Relevance to Matrix Biology. Matrix Biol. 2009, 28 (7), 384–389. 10.1016/j.matbio.2009.06.004. 19586615

[ref3] Humzah M. D.; Soames R. W. Human Intervertebral Disc: Structure and Function. Anat. Rec. 1988, 220 (4), 337–356. 10.1002/ar.1092200402. 3289416

[ref4] Trout J. J.; Buckwalter J. A.; Moore K. C. Ultrastructure of the Human Intervertebral Disc: II. Cells of the Nucleus Pulposus. Anat. Rec. 1982, 204 (4), 307–314. 10.1002/ar.1092040403. 7181135

[ref5] Risbud M. V.; Schaer T. P.; Shapiro I. M. Toward an Understanding of the Role of Notochordal Cells in the Adult Intervertebral Disc: From Discord to Accord. Dev. Dyn. 2010, 239 (8), 2141–2148. 10.1002/dvdy.22350. 20568241 PMC3634351

[ref6] Gantenbein B.; Calandriello E.; Wuertz-Kozak K.; Benneker L. M.; Keel M. J. B.; Chan S. C. W. Activation of Intervertebral Disc Cells by Co-Culture with Notochordal Cells, Conditioned Medium and Hypoxia. BMC Musculoskelet. Disord. 2014, 15 (1), 1–15. 10.1186/1471-2474-15-422. 25496082 PMC4295479

[ref7] de Vries S. A. H.; Potier E.; van Doeselaar M.; Meij B. P.; Tryfonidou M. A.; Ito K. Conditioned Medium Derived from Notochordal Cell-Rich Nucleus Pulposus Tissue Stimulates Matrix Production by Canine Nucleus Pulposus Cells and Bone Marrow-Derived Stromal Cells. Tissue Eng. Part A 2015, 21 (5–6), 1077–1084. 10.1089/ten.tea.2014.0309. 25370929 PMC4356475

[ref8] de Vries S. A. H.; van Doeselaar M.; Meij B. P.; Tryfonidou M. A.; Ito K. The Stimulatory Effect of Notochordal Cell-Conditioned Medium in a Nucleus Pulposus Explant Culture. Tissue Eng. Part A 2016, 22 (1–2), 103–110. 10.1089/ten.tea.2015.0121. 26421447

[ref9] Bach F.; de Vries S.; Krouwels A; Creemers L.; Ito K; Meij B.; Tryfonidou M. The Species-Specific Regenerative Effects of Notochordal Cell-Conditioned Medium on Chondrocyte-like Cells Derived from Degenerated Human Intervertebral Discs. Eur. Cells Mater. 2015, 30 (2015), 132–147. 10.22203/eCM.v030a10. 26388616

[ref10] Trout J. J.; Buckwalter J. A.; Moore K. C.; Landas S. K. Ultrastructure of the Human Intervertebral Disc. I. Changes in Notochordal Cells with Age. Tissue Cell 1982, 14 (2), 359–369. 10.1016/0040-8166(82)90033-7. 7202266

[ref11] Risbud M. V.; Shapiro I. M. Notochordal Cells in the Adult Intervertebral Disc: New Perspective on an Old Question. Crit. Rev. Eukaryot. Gene Expr. 2011, 21 (1), 29–41. 10.1615/CritRevEukarGeneExpr.v21.i1.30. 21967331 PMC3187872

[ref12] McCann M.; Séguin C. Notochord Cells in Intervertebral Disc Development and Degeneration. J. Dev. Biol. 2016, 4 (1), 3 10.3390/jdb4010003. 27252900 PMC4885739

[ref13] Bach F.; Libregts S.; Creemers L.; Meij B.; Ito K.; Wauben M.; Tryfonidou M. Notochordal-Cell Derived Extracellular Vesicles Exert Regenerative Effects on Canine and Human Nucleus Pulposus Cells. Oncotarget 2017, 8 (51), 88845–88856. 10.18632/oncotarget.21483. 29179481 PMC5687651

[ref14] Cornejo M. C.; Cho S. K.; Giannarelli C.; Iatridis J. C.; Purmessur D. Soluble Factors from the Notochordal-Rich Intervertebral Disc Inhibit Endothelial Cell Invasion and Vessel Formation in the Presence and Absence of pro-Inflammatory Cytokines. Osteoarthr. Cartil. 2015, 23 (3), 487–496. 10.1016/j.joca.2014.12.010. PMC4411226 25534363

[ref15] Matta A.; Karim M. Z.; Isenman D. E.; Erwin W. M. Molecular Therapy for Degenerative Disc Disease: Clues from Secretome Analysis of the Notochordal Cell-Rich Nucleus Pulposus. Sci. Rep. 2017, 7, 1–14. 10.1038/srep45623. 28358123 PMC5372366

[ref16] Bai X.-D.; Li X.-C.; Chen J.-H.; Guo Z.-M.; Hou L.-S.; Wang D.-L.; He Q.; Ruan D.-K. Coculture with Partial Digestion Notochordal Cell-Rich Nucleus Pulposus Tissue Activates Degenerative Human Nucleus Pulposus Cells. Tissue Eng. Part A 2017, 23 (15–16), 837–846. 10.1089/ten.tea.2016.0428. 28145804

[ref17] Bach F.; de Vries S.; Riemers F.; Boere J; van Heel F.; van Doeselaar M; Goerdaya S.; Nikkels P.; Benz K; Creemers L.; et al. Soluble and Pelletable Factors in Porcine, Canine and Human Notochordal Cell-Conditioned Medium: Implications for IVD Regeneration. Eur. Cells Mater. 2016, 32, 163–180. 10.22203/eCM.v032a11. 27572543

[ref18] Mwale F; Roughley P; Antoniou J Distinction between the Extracellular Matrix of the Nucleus Pulposus and Hyaline Cartilage: A Requisite for Tissue Engineering of Intervertebral Disc. Eur. Cells Mater. 2004, 8, 58–64. 10.22203/eCM.v008a06. 15602703

[ref19] De Vries S. A. H.; Van Doeselaar M.; Kaper H. J.; Sharma P. K.; Ito K. Notochordal Cell Matrix as a Bioactive Lubricant for the Osteoarthritic Joint. Sci. Rep. 2018, 8 (1), 8875 10.1038/s41598-018-27130-9. 29891965 PMC5995895

[ref20] Bach F. C.; Tellegen A. R.; Beukers M.; Miranda-Bedate A.; Teunissen M.; de Jong W. A. M.; de Vries S. A. H.; Creemers L. B.; Benz K.; Meij B. P.; Ito K.; Tryfonidou M. A. Biologic Canine and Human Intervertebral Disc Repair by Notochordal Cell-Derived Matrix: From Bench towards Bedside. Oncotarget 2018, 9 (41), 26507–26526. 10.18632/oncotarget.25476. 29899873 PMC5995168

[ref21] Wilson C. A. Endogenous Retroviruses. Cell. Mol. Life Sci. 2008, 65 (21), 3399–3412. 10.1007/s00018-008-8498-z. 18818871 PMC11131834

[ref22] Martin U.; Winkler M. E.; Id M.; Radeke H.; Arseniev L.; Takeuchi Y.; Simon A. R.; Patience C.; Haverich A.; Steinhoff G. Productive Infection of Primary Human Endothelial Cells by Pig Endogenous Retrovirus (PERV). Xenotransplantation 2000, 7 (2), 138–142. 10.1034/j.1399-3089.2000.00052.x. 10961298

[ref23] Poli C.; Augusto J. F.; Dauvé J.; Adam C.; Preisser L.; Larochette V.; Pignon P.; Savina A.; Blanchard S.; Subra J. F.; Chevailler A.; Procaccio V.; Croué A.; Créminon C.; Morel A.; Delneste Y.; Fickenscher H.; Jeannin P. IL-26 Confers Proinflammatory Properties to Extracellular DNA. J. Immunol. 2017, 198 (9), 3650–3661. 10.4049/jimmunol.1600594. 28356384

[ref24] Motwani M.; Pesiridis S.; Fitzgerald K. A. DNA Sensing by the CGAS–STING Pathway in Health and Disease. Nat. Rev. Genet. 2019, 20 (11), 657–674. 10.1038/s41576-019-0151-1. 31358977

[ref25] Nagata S.; Hanayama R.; Kawane K. Autoimmunity and the Clearance of Dead Cells. Cell 2010, 140 (5), 619–630. 10.1016/j.cell.2010.02.014. 20211132

[ref26] Mercuri J. J.; Gill S. S.; Simionescu D. T. Novel Tissue-Derived Biomimetic Scaffold for Regenerating the Human Nucleus Pulposus. J. Biomed. Mater. Res. - Part A 2011, 96A (2), 422–435. 10.1002/jbm.a.33001. 21171162

[ref27] Wachs R. A.; Hoogenboezem E. N.; Huda H. I.; Xin S.; Porvasnik S. L.; Schmidt C. E. Creation of an Injectable in Situ Gelling Native Extracellular Matrix for Nucleus Pulposus Tissue Engineering. Spine J. 2017, 17 (3), 435–444. 10.1016/j.spinee.2016.10.022. 27989725

[ref28] Xu J.; Liu S.; Wang S.; Qiu P.; Chen P.; Lin X.; Fang X. Decellularised Nucleus Pulposus as a Potential Biologic Scaffold for Disc Tissue Engineering. Mater. Sci. Eng., C 2019, 99, 1213–1225. 10.1016/j.msec.2019.02.045. 30889657

[ref29] Gratzer P. F.; Harrison R. D.; Woods T. Matrix Alteration and Not Residual Sodium Dodecyl Sulfate Cytotoxicity Affects the Cellular Repopulation of a Decellularized Matrix. Tissue Eng. 2006, 12 (10), 2975–2983. 10.1089/ten.2006.12.2975. 17518665

[ref30] Cebotari S.; Tudorache I.; Jaekel T.; Hilfiker A.; Dorfman S.; Ternes W.; Haverich A.; Lichtenberg A. Detergent Decellularization of Heart Valves for Tissue Engineering: Toxicological Effects of Residual Detergents on Human Endothelial Cells. Artif. Organs 2010, 34 (3), 206–210. 10.1111/j.1525-1594.2009.00796.x. 20447045

[ref31] Gilbert T. W.; Sellaro T. L.; Badylak S. F. Decellularization of Tissues and Organs. Biomaterials 2006, 27 (19), 3675–3683. 10.1016/j.biomaterials.2006.02.014. 16519932

[ref32] Faulk D. M.; Carruthers C. A.; Warner H. J.; Kramer C. R.; Reing J. E.; Zhang L.; D’Amore A.; Badylak S. F. The Effect of Detergents on the Basement Membrane Complex of a Biologic Scaffold Material. Acta Biomater. 2014, 10 (1), 183–193. 10.1016/j.actbio.2013.09.006. 24055455 PMC3857635

[ref33] Keane T. J.; Londono R.; Turner N. J.; Badylak S. F. Consequences of Ineffective Decellularization of Biologic Scaffolds on the Host Response. Biomaterials 2012, 33 (6), 1771–1781. 10.1016/j.biomaterials.2011.10.054. 22137126

[ref34] White L. J.; Taylor A. J.; Faulk D. M.; Keane T. J.; Saldin L. T.; Reing J. E.; Swinehart I. T.; Turner N. J.; Ratner B. D.; Badylak S. F. The Impact of Detergents on the Tissue Decellularization Process: A ToF-SIMS Study. Acta Biomater. 2017, 50, 207–219. 10.1016/j.actbio.2016.12.033. 27993639 PMC5592694

[ref35] Galili U. The α-Gal Epitope (Galα1–3Galβ1–4GlcNAc-R) in Xenotransplantation. Biochimie 2001, 83 (7), 557–563. 10.1016/S0300-9084(01)01294-9. 11522383

[ref36] Galili U. Macrophages Recruitment and Activation by α-Gal Nanoparticles Accelerate Regeneration and Can Improve Biomaterials Efficacy in Tissue Engineering. Open Tissue Eng. Regen. Med. J. 2013, 6 (1), 1–11. 10.2174/1875043501306010001.

[ref37] Piening L. M.; Lillyman D. J.; Lee F. S.; Lozano A. M.; Miles J. R.; Wachs R. A. Injectable Decellularized Nucleus Pulposus Tissue Exhibits Neuroinhibitory Properties. JOR Spine 2022, 2021, 1–16. 10.1002/jsp2.1187. PMC8966883 35386760

[ref38] Schmitz T. C.; Dede Eren A.; Spierings J.; Boer J.; Ito K.; Foolen J. Solid-Phase Silica-Based Extraction Leads to Underestimation of Residual DNA in Decellularized Tissues. Xenotransplantation 2021, 28 (1), 1–7. 10.1111/xen.12643. PMC9286341 32935355

[ref39] Wall A.; Board T. A Direct Spectrophotometric Microassay for Sulphated Glycosaminoglycans in Cartilage Cultures. Class. Pap. Orthop. 2014, 9, 431–432. 10.1007/978-1-4471-5451-8_109.

[ref40] Huszar G.; Maiocco J.; Naftolin F. Monitoring of Collagen and Collagen Fragments in Chromatography of Protein Mixtures. Anal. Biochem. 1980, 105 (1), 424–429. 10.1016/0003-2697(80)90481-9. 7457846

[ref41] Bach F. C.; Laagland L. T.; Grant M. P.; Creemers L. B.; Ito K.; Meij B. P.; Mwale F.; Tryfonidou M. A. Link-N: The Missing Link towards Intervertebral Disc Repair Is Species-Specific. PLoS One 2017, 12 (11), e0187831 10.1371/journal.pone.0187831. 29117254 PMC5679057

[ref42] de Vries S.; van Doeselaar M.; Meij B.; Tryfonidou M.; Ito K. Notochordal Cell Matrix As a Therapeutic Agent for Intervertebral Disc Regeneration. Tissue Eng. Part A 2019, 25 (11–12), 830–841. 10.1089/ten.tea.2018.0026. 29739272

[ref43] Livak K. J.; Schmittgen T. D. Analysis of Relative Gene Expression Data Using Real-Time Quantitative PCR and the 2−ΔΔCT Method. Methods 2001, 25 (4), 402–408. 10.1006/meth.2001.1262. 11846609

[ref44] Hofmann S.; Hagenmüller H.; Koch A. M.; Müller R.; Vunjak-Novakovic G.; Kaplan D. L.; Merkle H. P.; Meinel L. Control of in Vitro Tissue-Engineered Bone-like Structures Using Human Mesenchymal Stem Cells and Porous Silk Scaffolds. Biomaterials 2007, 28 (6), 1152–1162. 10.1016/j.biomaterials.2006.10.019. 17092555

[ref45] Van Dijk B.; Potier E.; Ito K. Culturing Bovine Nucleus Pulposus Explants by Balancing Medium Osmolarity. Tissue Eng. - Part C Methods 2011, 17 (11), 1089–1096. 10.1089/ten.tec.2011.0215. 21718168

[ref46] Fernandez C.; Marionneaux A.; Gill S.; Mercuri J. Biomimetic Nucleus Pulposus Scaffold Created from Bovine Caudal Intervertebral Disc Tissue Utilizing an Optimal Decellularization Procedure. J. Biomed. Mater. Res. - Part A 2016, 104 (12), 3093–3106. 10.1002/jbm.a.35858. PMC5832047 27507100

[ref47] Gilbert T. W.; Freund J. M.; Badylak S. F. Quantification of DNA in Biologic Scaffold Materials. J. Surg. Res. 2009, 152 (1), 135–139. 10.1016/j.jss.2008.02.013. 18619621 PMC2783373

[ref48] Filippo N.; Paola A.; Laura I. Biocompatibility Evaluation Criteria for Novel Xenograft Materials: Distribution and Quantification of Remnant Nucleic Acid and Alpha-Gal Epitope. J. Stem Cell Res. Ther. 2013, 01 ( (S6), ), 10.4172/2157-7633.S6-009.

[ref49] Frank M. O. Circulating Cell-Free DNA Differentiates Severity of Inflammation. Biol. Res. Nurs. 2016, 18 (5), 477–488. 10.1177/1099800416642571. 27067611

[ref50] Maroudas A.; Stockwell R. A.; Nachemson A.; Urban J. Factors Involved in the Nutrition of the Human Lumbar Intervertebral Disc: Cellularity and Diffusion of Glucose in Vitro. J. Anat. 1975, 120 (Pt 1), 113–130. 1184452 PMC1231728

[ref51] Czauderna F.; Fischer N.; Boller K.; Kurth R.; Tonjes R. R. Establishment and Characterization of Molecular Clones of Porcine Endogenous Retroviruses Replicating on Human Cells. J. Virol. 2000, 74 (9), 4028–4038. 10.1128/JVI.74.9.4028-4038.2000. 10756014 PMC111916

[ref52] Lopata K.; Wojdas E.; Nowak R.; Lopata P.; Mazurek U. Porcine Endogenous Retrovirus (PERV)-Molecular Structure and Replication Strategy in the Context of Retroviral Infection Risk of Human Cells. Front. Microbiol. 2018, 9 (APR), 1–11. 10.3389/fmicb.2018.00730. 29755422 PMC5932395

[ref53] Mercuri J. J.; Patnaik S.; Dion G.; Gill S. S.; Liao J.; Simionescu D. T. Regenerative Potential of Decellularized Porcine Nucleus Pulposus Hydrogel Scaffolds: Stem Cell Differentiation, Matrix Remodeling, and Biocompatibility Studies. Tissue Eng. Part A 2013, 19 (7–8), 952–966. 10.1089/ten.tea.2012.0088. 23140227

[ref54] Wang M.; Liu X.; Lyu Z.; Gu H.; Li D.; Chen H. Glycosaminoglycans (GAGs) and GAG Mimetics Regulate the Behavior of Stem Cell Differentiation. Colloids Surfaces B Biointerfaces 2017, 150, 175–182. 10.1016/j.colsurfb.2016.11.022. 27914254

[ref55] Heino J. The Collagen Family Members as Cell Adhesion Proteins. BioEssays 2007, 29 (10), 1001–1010. 10.1002/bies.20636. 17876790

[ref56] Xiang L.; Chen K.; Yan R.; Li W.; Xu K. Single-Molecule Displacement Mapping Unveils Nanoscale Heterogeneities in Intracellular Diffusivity. Nat. Methods 2020, 17 (5), 524–530. 10.1038/s41592-020-0793-0. 32203387 PMC7205592

[ref57] Nesmelova I. V.; Melnikova D. L.; Ranjan V.; Skirda V. D. Translational Diffusion of Unfolded and Intrinsically Disordered Proteins, 1st ed.; Elsevier, 2019; Vol. 166. 10.1016/bs.pmbts.2019.05.004 31521238

[ref58] Schavemaker P. E.; Boersma A. J.; Poolman B. How Important Is Protein Diffusion in Prokaryotes?. Front. Mol. Biosci. 2018, 5 (NOV), 1–16. 10.3389/fmolb.2018.00093. 30483513 PMC6243074

[ref59] Abaskharon R. M.; Gai F. Direct Measurement of the Tryptophan-Mediated Photocleavage Kinetics of a Protein Disulfide Bond. Phys. Chem. Chem. Phys. 2016, 18 (14), 9602–9607. 10.1039/C6CP00865H. 26997094 PMC4814302

[ref60] Durchschlag H.; Fochler C.; Feser B.; Hausmann S.; Seroneit T.; Swientek M.; Swoboda E.; Winklmair A.; Wlček C.; Zipper P. Effects of X-and UV-Irradiation on Proteins. Radiat. Phys. Chem. 1996, 47 (3), 501–505. 10.1016/0969-806X(95)00138-N.

[ref61] Johnson Z. I.; Schoepflin Z. R.; Choi H.; Shapiro I. M.; Risbud M. V. Disc in Flames: Roles of TNF-α and IL-1β in Intervertebral Disc Degeneration. Eur. Cell. Mater. 2015, 30, 104–117. 10.22203/eCM.v030a08. 26388614 PMC4751407

[ref62] Cortes D. H.; Jacobs N. T.; Delucca J. F.; Elliott D. M. Elastic, Permeability and Swelling Properties of Human Intervertebral Disc Tissues : A Benchmark for Tissue Engineering. J. Biomech. 2014, 47 (9), 2088–2094. 10.1016/j.jbiomech.2013.12.021. 24438768 PMC4047194

[ref63] Saldin L. T.; Cramer M. C.; Velankar S. S.; White L. J.; Badylak S. F. Extracellular Matrix Hydrogels from Decellularized Tissues: Structure and Function. Acta Biomater. 2018, 1 (49), 1–15. 10.1016/j.actbio.2016.11.068.Extracellular. PMC5253110 27915024

[ref64] Johnston N.; Dettmar P. W.; Bishwokarma B.; Lively M. O.; Koufman J. A. Activity/Stability of Human Pepsin: Implications for Reflux Attributed Laryngeal Disease. Laryngoscope 2007, 117 (6), 1036–1039. 10.1097/MLG.0b013e31804154c3. 17417109

[ref65] Piper D. W.; Fenton B. H. PH Stability and Activity Curves of Pepsin with Special Reference to Their Clinical Importance. Gut 1965, 6 (5), 506–508. 10.1136/gut.6.5.506. 4158734 PMC1552331

[ref66] Hamuro Y.; Coales S. J.; Molnar K. S.; Tuske S. J.; Morrow J. A. Specificity of Immobilized Porcine Pepsin in H/D Exchange Compatible Conditions. Rapid Commun. Mass Spectrom. 2008, 22 (7), 1041–1046. 10.1002/rcm.3467. 18327892

[ref67] Metzner A. B. Rheology of Suspensions in Polymeric Liquids. J. Rheol. (N. Y. N. Y). 1985, 29 (6), 739–775. 10.1122/1.549808.

[ref68] Wilkes G. L. An Overview of the Basic Rheological Behavior of Polymer Fluids with an Emphasis on Polymer Melts. J. Chem. Educ. 1981, 58 (11), 880–892. 10.1021/ed058p880.

[ref69] Johannessen W.; Elliott D. M. Effects of Degeneration on the Biphasic Material Properties of Human Nucleus Pulposus in Confined Compression. Spine (Phila. Pa. 1976) 2005, 30 (24), E724–9. 10.1097/01.brs.0000192236.92867.15. 16371889

[ref70] Schmitz T. C.; Salzer E.; Crispim J. F.; Fabra G. T.; LeVisage C.; Pandit A.; Tryfonidou M.; Maitre C. Le; Ito K. Characterization of Biomaterials Intended for Use in the Nucleus Pulposus of Degenerated Intervertebral Discs. Acta Biomater. 2020, 114, 1–15. 10.1016/j.actbio.2020.08.001. 32771592

[ref71] Aguado B. A.; Mulyasasmita W.; Su J.; Lampe K. J.; Heilshorn S. C. Improving Viability of Stem Cells during Syringe Needle Flow through the Design of Hydrogel Cell Carriers. Tissue Eng. - Part A 2012, 18 (7–8), 806–815. 10.1089/ten.tea.2011.0391. 22011213 PMC3313609

[ref72] Loibl M.; Wuertz-Kozak K.; Vadala G.; Lang S.; Fairbank J.; Urban J. P. Controversies in Regenerative Medicine: Should Intervertebral Disc Degeneration Be Treated with Mesenchymal Stem Cells. Jor Spine 2019, 2 (1), e1043 10.1002/jsp2.1043. 31463457 PMC6711491

[ref73] Clouet J.; Fusellier M.; Camus A.; Le Visage C.; Guicheux J. Intervertebral Disc Regeneration: From Cell Therapy to the Development of Novel Bioinspired Endogenous Repair Strategies. Adv. Drug Delivery Rev. 2019, 146, 306–324. 10.1016/j.addr.2018.04.017. 29705378

[ref74] Content and Review of Chemistry, Manufacturing, and Control (CMC) Information for Human Somatic Cell Therapy Investigational New Drug Applications (INDs): Guidance for FDA Reviewers and Sponsors; U.S. Food and Drug Administration, 2008; pp 1–36.

[ref75] Kondziolka D.; Gobbel G. T.; Fellows-Mayle W.; Chang Y. F.; Uram M. Injection Parameters Affect Cell Viability and Implant Volumes in Automated Cell Delivery for the Brain. Cell Transplant. 2011, 20 (11–12), 1901–1906. 10.3727/096368911X566190. 21457614

[ref76] Walker P. A.; Jimenez F.; Aroom K.; Gill B. S.; Savitz S. I.; Cox C. S. Effect of Needle Diameter and Flow Rate on Mesenchymal Stromal Cell (MSC) Cell Characterization and Viability. J. Surg. Res. 2010, 158 (2), 382 10.1016/j.jss.2009.11.581. PMC2943405 20001789

[ref77] Chen J.; Mei Z.; Huang B.; Zhang X.; Liu J.; Shan Z.; Wang J.; Wang X.; Zhao F. IL-6/YAP1/β-Catenin Signaling Is Involved in Intervertebral Disc Degeneration. J. Cell. Physiol. 2019, 234 (5), 5964–5971. 10.1002/jcp.27065. 30511395 PMC6686169

[ref78] Dalgliesh A. J.; Parvizi M.; Lopera-Higuita M.; Shklover J.; Griffiths L. G. Graft-Specific Immune Tolerance Is Determined by Residual Antigenicity of Xenogeneic Extracellular Matrix Scaffolds. Acta Biomater. 2018, 79, 253–264. 10.1016/j.actbio.2018.08.016. 30130615 PMC6349227

[ref79] Galili U. Acceleration of Wound Healing by α-Gal Nanoparticles Interacting with the Natural Anti-Gal Antibody. J. Immunol. Res. 2015, 2015, 1–13. 10.1155/2015/589648. PMC4397477 25922849

[ref80] Lu Y.; Shao A.; Shan Y.; Zhao H.; Leiguo M.; Zhang Y.; Tang Y.; zhang W.; Jin Y.; Xu L. A Standardized Quantitative Method for Detecting Remnant Alpha-Gal Antigen in Animal Tissues or Animal Tissue-Derived Biomaterials and Its Application. Sci. Rep. 2018, 8 (1), 4–13. 10.1038/s41598-018-32959-1. 30337555 PMC6194003

